# RUNAWAY SEXUAL SELECTION WITHOUT GENETIC CORRELATIONS: SOCIAL ENVIRONMENTS AND FLEXIBLE MATE CHOICE INITIATE AND ENHANCE THE FISHER PROCESS

**DOI:** 10.1111/j.1558-5646.2012.01647.x

**Published:** 2012-09

**Authors:** Nathan W Bailey, Allen J Moore

**Affiliations:** 1 Centre for Biological Diversity, School of Biology, University of St AndrewsFife KY16 9TH, United Kingdom; 3 Centre for Ecology and Conservation, University of ExeterCornwall Campus, Penryn TR10 9EZ, United Kingdom; 4 Department of Genetics, University of GeorgiaAthens, Georgia 20602

**Keywords:** Fisherian runaway, indirect genetic effects, interacting phenotypes, mate choice plasticity, mate preference learning, social evolution

## Abstract

Female mating preferences are often flexible, reflecting the social environment in which they are expressed. Associated indirect genetic effects (IGEs) can affect the rate and direction of evolutionary change, but sexual selection models do not capture these dynamics. We incorporate IGEs into quantitative genetic models to explore how variation in social environments and mate choice flexibility influence Fisherian sexual selection. The importance of IGEs is that runaway sexual selection can occur in the absence of a genetic correlation between male traits and female preferences. Social influences can facilitate the initiation of the runaway process and increase the rate of trait elaboration. Incorporating costs to choice do not alter the main findings. Our model provides testable predictions: (1) genetic covariances between male traits and female preferences may not exist, (2) social flexibility in female choice will be common in populations experiencing strong sexual selection, (3) variation in social environments should be associated with rapid sexual trait divergence, and (4) secondary sexual traits will be more elaborate than previously predicted. Allowing feedback from the social environment resolves discrepancies between theoretical predictions and empirical data, such as why indirect selection on female preferences, theoretically weak, might be sufficient for preferences to become elaborated.

The social environment is arguably one of the most dynamic and influential sources of environmental variation an organism might experience during its lifetime (West-Eberhard 1983; Kent et al. 2008; Krupp et al. 2008). Social interactions are inherent in sexual reproduction, but their influence can extend beyond the immediate pairing of sexual partners to shape how females evaluate potential mates. Of particular note is when the attractiveness of a male trait to a female is enhanced or diminished by the wider social environment in which it is expressed. For example, empirical studies across a wide range of taxa have established that prior experience of male ornaments influences female preferences (Qvarnström et al. 2000; Hebets 2003; Bailey and Zuk 2009; Wong et al. 2011). These social effects on female choice can manifest in a variety of ways. Besides allowing for relative preferences, they can occur through sexual imprinting (Slagsvold et al. 2002), mate choice copying (Godin et al. 2005), context dependence (Royle et al. 2008), and learning (Svensson et al. 2010).

Quantitative genetic models of sexual selection have identified the primacy of genetic variance and covariance of ornaments and preferences for the elaboration of traits via a runaway process (Lande 1981; Kirkpatrick 1982; Mead and Arnold 2004), but feedback from the social environment has not been integrated in these models despite its potentially key role. Because such feedback is critical to the model we develop below, it is essential to provide a disciplined definition of “social feedback”: we define this to mean that the expression of female preferences varies depending on the male genotypes that they experience. The field cricket *Teleogryllus oceanicus* provides a specific empirical example. A mutant wing morphology, called *flatwing*, eliminates the ability of males to sing in some populations (Zuk et al. 2006). The acoustic environment experienced by females developing in those populations depends largely on the proportion of flatwing alleles in the population; *flatwing* segregates as a sex-linked single-locus trait (Tinghitella 2007), so a greater proportion of *flatwing* alleles directly translates to a more silent environment owing to the greater relative abundance of silent males. Thus, the average acoustic experience a female has will be related to the genotypes of males in her environment. A number of studies have demonstrated that the acoustic environment that *T. oceanicus* females experience alters their choice of mates, suggesting that variation in the social environment has a considerable impact on the evolution of sexually selected traits (Bailey and Zuk 2008, 2009; Bailey 2011; Rebar et al. 2011). Labile mating preferences may reflect learning. In a wide variety of vertebrate and invertebrate species, it is becoming increasingly recognized that learned mate preferences reflect properties of the social environment in which females develop (see e.g., Verzijden and Rosenthal 2011). Such social effects appear to be more of a rule rather than an exception. For example, *Drosophila serrata* males alter their cuticular hydrocarbon profile in response to the genotype of interacting females (Petfield et al. 2005), and *D. melanogaster* males alter their cuticular hydrocarbon profile in response to the genotypes of males in their environment (Kent et al. 2008).

We model sexual selection using an interacting phenotype approach, which incorporates indirect genetic effects (IGEs) that occur when genetically influenced traits in one individual alter the phenotype of an interacting partner (Moore et al. 1997; McGlothlin et al. 2010). This framework provides a straightforward method of illuminating the evolutionary dynamics that arise when male traits alter female preferences: when male secondary sexual characters exhibit additive genetic variation, IGEs are expected to play an important role if female choice is affected by the social environment imparted by those male traits. IGE models demonstrate that social environments have a genetic basis, evolve, and provide an evolutionary feedback that affects the rate and direction of evolutionary change in phenotypes that are expressed in social interactions (Moore et al. 1997; McGlothlin et al. 2010).

Interacting phenotype models and subsequent empirical work have shown that understanding social effects changes how we view the evolution of social traits. For example, breeding programs for social traits in livestock animals produce stronger responses to selection when IGEs contributed by the social environment are taken into consideration (Rodenburg et al. 2010; Wade et al. 2010). Selection in domesticated chickens that manipulates the effects of the social environment reduces pecking behavior and cannibalism above and beyond direct selection on pecking (Rodenburg et al. 2008). However, IGE models that could provide similar insights in a sexual selection context are lacking. We develop such models here and provide testable predictions that refine our understanding of the social context, genetics, and evolution of secondary sexual characters.

## Modeling Sexual Selection and Social Environments

Consider a population of sexually reproducing diploid individuals containing males with trait *t* and females with preference *p*. Adopting standard quantitative genetic assumptions (Falconer and Mackay 1996; Mead and Arnold 2004):


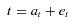
(1)

This is the simplest partitioning (Falconer and Mackay 1996) of the male trait into additive genetic effects, *a*, and all other (abiotic environmental and nonadditive genetic) effects, *e*. For simplicity, we assume that the male trait is a structure (such as a morphological trait) and its expression, for example, how large or showy it is (Darwin 1871), is unaffected by the social environment. In contrast, we allow the female preference, *p*, to potentially depend on the social environment in which it is expressed and therefore further partition the environmental term:


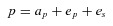
(2a)

Female preference describes the male trait value females prefer. By allowing the social environment to influence female preference, we can explore the effects of how specific males in a female's environment influence her preference. Social effects could influence female preference by changing the male trait value that females most prefer, or the degree to which they discriminate against nonpreferred trait values. The new term, *e_s_*, is the social environment provided by males, which is determined by the trait value of the males with whom the female interacts. This abstract definition of the social environment can again be tied to empirical examples such as the cricket one above. In that case, the distribution of male genotypes contributes to the acoustic environment that females experience during development, and variation in the acoustic environment causes corresponding changes in the expression of female preferences (Bailey and Zuk 2008, 2009; Bailey 2011; Rebar et al. 2011). For heuristic simplicity, we will consider only one interacting male and one interacting trait. Interactions with multiple males can be incorporated by substituting the mean social environment; that is, the mean value for all of the male traits, and evaluation of multiple traits by females can be achieved using a multivariate model (Moore et al. 1997). Neither of these changes the basic results.

Because the social environment reflects the male trait, we can set *e_s_*=Ψ*t*′, where *t*′ is the trait value, *t*, of a male interacting with a female. The prime indicates that the trait is expressed in a social partner (i.e., not the trait of a focal individual for whom the phenotype is being defined [Moore et al. 1997]). The impact of the social environment is scaled by the coefficient Ψ, which in theory can take on any value from –1 to 1, and reflects the relative importance of the social environment on the expression of a preference (Bleakley et al. 2010). The coefficient Ψ is analogous to m, the maternal effect coefficient in maternal effect models that describes the strength of the effect that the mother's phenotype has on determining the phenotype of offspring independent of direct genetic effects (Kirkpatrick and Lande 1989). Empirical measurements of Ψ have shown that it can be either negative or positive, but it is typically strong (Bleakley et al. 2010; Bailey and Zuk, in press). Being a mathematical constant, Ψ can be thought of as a population parameter as it is measured by the partial regression of the focal individual on interacting individual(s), holding their genetic make-up constant (Moore et al. 1997; Bleakley et al. 2010). We also discuss the role of this coefficient further below.

Substituting for *t*′ gives


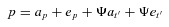
(2b)

Thus, the female preference is influenced by direct genetic, *a_p_*, and environmental, *e_p_*, effects, plus indirect additive genetic, 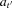, and environmental, 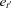, effects arising from male traits of the interacting individual. As with standard quantitative genetic models (Falconer and Mackay 1996), we assume no covariance between additive genetic effects and environmental effects; likewise, the present model assumes that environmental effects and IGEs contributed by the interacting partner, 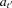, are independent.

**Figure 1:**
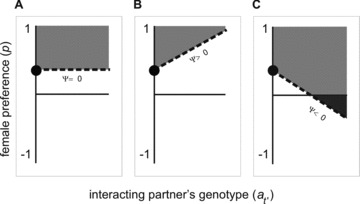
The interaction coefficient Ψ. The genotypic range of a male trait that influences preferences, 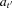, is portrayed on the *x*-axis. The *y*-axis shows female preference, *p*, defined as the difference between the male trait value she chooses and the average male trait, 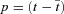. The inherent preference of females is the *y*-intercept given by *a_p_* (solid circle). We illustrate females with inherent preferences for greater than average male trait values, which should be prevalent under directional and open-ended preferences. The change in acceptable trait values (dashed line) is dictated by Ψ. (a) Ψ= 0. Female preference does not change across the range of male phenotypes in the social environment. (b) Ψ > 0. Social interactions with larger male traits increase female preferences. (c) Ψ < 0. Social experience decreases female preference. The light shaded areas above the dashed lines indicate the male trait values, relative to the average, that a female will accept given her inherent preference and the social environment experienced. Dark shading in (c) indicates conditions in which females accept below average males.

Social environments can exert positive or negative influences, and in the context of sexual selection the coefficient Ψ describes the degree to which female preference is enhanced or diminished as a result of interacting with a male trait *t*′ (Fig. 1). This could occur through changes in the values of male traits that females prefer, as has been found in many birds and in wolf spiders (ten Cate and Vos 1999; Hebets 2003), or the strength with which that preference is exercised, as has been shown in crickets and treehoppers (Bailey and Zuk 2009; Fowler-Finn and Rodriguez 2012). For example, Ψ is positive if females learn about the availability and quality of males around them and then leverage that information against their existing preferences to choose a better male. The field cricket system discussed above provides an empirical example of Ψ; within a population, females increase their choosiness after experiencing more attractive male songs (Bailey and Zuk 2009; Rebar et al. 2011), which means that Ψ is positive. An example of negative Ψ would be if, on average, females become less choosy as a result of social interactions, such as when female mate choice is abandoned in the face of strong male–male competition. As such, Ψ is determined by the population of individual interactions. It is conceptually and mechanistically distinct from the evolving traits under consideration, reflecting the effect of an interaction, rather than the traits expressed in the interaction, which in this instance are male ornaments and female preferences.

Evolution depends on the nature of selection as well as genetics. To model selection, we adapt standard definitions of fitness (Iwasa and Pomiankowski 1995), where fitness associated with natural selection on the male trait (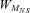) centers around an optimum and fitness associated with sexual selection (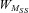) reflects a preference for greater-than-average trait expression


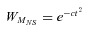
(3a)


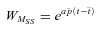
(3b)

A male's fitness depends on his trait value, *t*, and the natural selection cost, *c*, of this value (eq. 3a). The trait also influences fitness through mating (sexual selection; eq. 3b), where fitness associated with the trait depends on the average female preference, 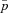. The constant *a* determines the steepness of the relationship between female preference and male fitness. We assume a model of preference most consistent with the existing data: preference is scaled by the relative deviation of the male trait value from the average male trait value in the population (Lande 1981). From the fitness equations, we can generate selection gradients by taking the partial derivatives evaluated at the population means 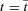 and 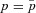.

Given these equations, evolutionary change in *t* and *p* can be estimated by examining the action of selection on the covariance between the breeding value and phenotypic value for each trait (Lande 1981). However, unlike the male trait *t*, female preference *p* is influenced by more than direct additive effects; its breeding value will include the IGEs contributed by the interacting trait scaled by Ψ (eq. 2b) (Moore et al. 1997). We begin by assuming that there is no selection on preferences, which would be true at the outset of selection (Kirkpatrick and Barton 1997). Later we relax this assumption. Again making standard quantitative genetic assumptions (Falconer and Mackay 1996; Mead and Arnold 2004) including constant additive genetic variances and covariances, the joint evolution of the male and female characters is


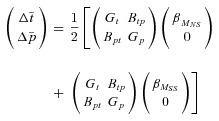
(4)

The 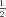 reflects the sex-limited expression of both traits, *G_t_* and *G_p_* are the additive genetic variances for *t* and *p*, respectively, and *B_tp_* is the genetic covariance. There is also sex-specific selection (β), which can be either directional sexual selection or stabilizing natural selection. Other forms of selection are possible, such as directional natural selection and stabilizing sexual selection, but we restrict the forms of selection to those that are expected to result in extreme trait elaboration via sexual selection (sensu Darwin 1871).

Changes in mean male trait values and mean female preferences are examined by evaluating these covariances at the population means 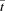 and 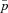 and simplifying


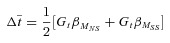
(5)


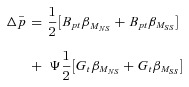
(6)

Equations (5) and (6) give an interacting phenotype model of sexual selection.

### INTERPRETATION: THE BASIC MODEL

**Figure 2:**
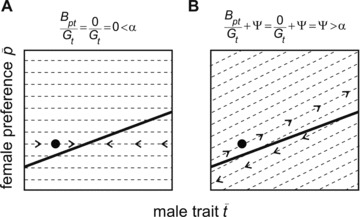
The effect of IGEs on the likelihood of runaway sexual selection. The solid circle represents a population that has been displaced slightly from a line of equilibrium with positive slope α, indicated by the heavy line. The dashed lines indicate the slope of the population's evolutionary trajectory as derived in the main text and indicated above the graphs. Both graphs show a situation in which covariance between trait *t* and preference *p* is nonexistent. The direction of evolution is indicated by small arrows. Traits lacking IGEs (i.e., when Ψ= 0) are shown in (a). The male trait evolves to a stable optimum due to the action of natural selection, and female preferences do not change except by drift. (b) The social environment provided by male traits affects expression of female preference. If Ψ exceeds α, the population evolves via runaway along a slope and in a direction indicated by the dashed lines. Once a covariance develops, runaway is enhanced.

The influence of IGEs that arise when female preference expression reflects the social environment is clear. The first half of equation (6) shows that the change in average female preference is a function of selection acting on females through genetic covariance with males, as predicted by Fisher (1958), modeled by Lande (1981) and confirmed by others (Kirkpatrick 1982; Pomiankowski et al. 1991; Iwasa and Pomiankowski 1995; Hall et al. 2000; Mead and Arnold 2004). However, the second half shows that preferences can also change as a result of selection acting on the male trait, filtered through Ψ, when there are IGEs. The social environment therefore has a genetic basis, it can evolve, and this evolution feeds back to affect the trait influenced by social context. As a result, female preferences can also evolve independently of a covariance between direct genetic effects. This does not diminish the importance of the covariance. Rather, it shows that a covariance between direct additive effects of the preference and the male trait can be sufficient but is not necessary for runaway evolution (Fig. 2).

The ability to achieve runaway in the absence of a genetic covariance has several profound effects on the likelihood of elaboration by sexual selection. One of the criticisms of the original model of the Fisher runaway process is that it depends on indirect selection for the evolution of female mating preferences (Kirkpatrick and Barton 1997) because selection on preferences acts through the genetic covariance, which effectively weakens the extent of evolutionary change. This theoretical difficulty disappears in our model. Given a sufficiently strong Ψ, female preference can evolve identically to the male trait even in the absence of direct genetic effects on the preference (eqs. 5 and 6). Male sexual signals and displays often occur in social contexts such as leks or mating rendezvous, and under these conditions the social environment might be particularly important (a strong positive Ψ) thereby facilitating runaway simply because of the importance of the male trait evolution for both characters. This enhancement of the joint evolution does not rely on a measurable genetic covariance between the direct additive genetic effects of the male trait and the female preference, which may explain why a surprising number of studies have found lower than expected—or no—covariances, as in jungle fowl (*Gallus gallus*) (Johnson et al. 1993), collared flycatchers (*Ficedula albicollis*) (Qvarnström et al. 2006), and the fruit fly *D. montana* (Ritchie et al. 2005). Also consistent with IGEs, the buildup of covariance has been found to be heterogeneous and dependent on environmental factors in other systems (Jia and Greenfield 1997).

## Initiation of Runaway

Because the genetic covariance between additive effects on male traits and female preferences is not required for the joint evolution of the traits, our model also suggests a pivotal role for social influences in the initiation of the runaway process. Fisher (1915, 1958) provided a verbal model for the origin of the runaway. It is worth quoting him in full:

“The most difficult and important act of choice is the choice of a mate; and this would have been rendered possible in the first instance by focussing the mind, as yet unable to make any profound judgement, upon certain conspicuous points which readily attract attention, and which attain by Natural Selection an innate prejudice in their favour.” (Fisher 1915, p. 186)

Fisher's suggestion was that male traits that eventually become elaborated through the runaway process initially confer a small natural selection advantage to their bearer and are noticed by females; in other words, they allow the male to stand out in a crowd. Our model provides an analytical solution for this origin. Initially, there will be no genetic covariance between the male trait and female preference. Sexual selection on the male trait is nonexistent. Thus, the only selection acting on the male trait arises from natural selection


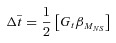
(7)

However, even in the absence of the genetic covariance between *t* and *p*, 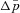 can be positive because of the social effects of the male trait. Setting *B_pt_*= 0 and 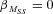 in equation (6):


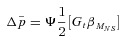
(8)

### INTERPRETATION: INITIATION OF RUNAWAY

A small natural selection advantage for the male trait results in a positive 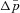 provided that Ψ > 0. The social environment thereby provides a mechanism for initiating the Fisher process when there is only natural selection on the male trait, prior to the buildup of genetic covariance. However, IGEs mediated through social environments can push the two initially uncorrelated traits closer toward a coevolutionary trajectory, resulting in faster runaway and buildup of a covariance. Social effects that might accomplish this correspond to Fisher's (1915) original suggestion for factors that initiate runaway. For example, social interactions could make it easier for females to find a mate, through a sharpening or tuning of perceptual ability, or in Fisher's (1915, p. 185) words: “[t]he task of determining the different qualities and abilities needed for biological success, and of recognising and weighing them within a short acquaintanceship…by the keenest observation.”

A number of systems provide empirical examples of how social interactions might “focus the mind” of choosing individuals. Learning could play a role. In several role-reversed damselfly species, for example, males lack innate preferences for female color morphs, but males show a learned sensory bias toward one or the other morph depending on prior experience with the different morphs (Fincke et al. 2007; Gosden and Svensson 2008, 2009; Takahashi and Watanabe 2010). During the early stages of trait and preference coevolution, these and other social effects that enhance the expression of preferences could contribute IGEs that attenuate the covariance required to generate unstable runaway conditions.

## Conditions Favoring Runaway

The second step in the Fisher process is the runaway itself; the exponential evolutionary increase in traits that occurs during the coevolution of male ornaments and female preferences (Mead and Arnold 2004). A robust definition for the conditions under which Fisherian runaway occurs was one of the most influential results of Lande's (1981) original sexual selection model. In his model, populations evolve toward a line of equilibrium in the case of a stable equilibrium, or exponentially away from the line in the case of an unstable equilibrium. Evolution away from the line of equilibrium results in rapid exaggeration or diminution of the trait and preference, and the runaway process refers to this unstable condition of rapid evolution. The stability of the equilibrium depends on its slope relative to the evolutionary trajectory of the population. When the population trajectory exceeds the slope of the line of equilibrium, the equilibrium is unstable and runaway occurs. The evolutionary trajectory of the population is described by the rate of change in female preference relative to the rate of change in the male trait, or the genetic covariance between trait and preference, *B_pt_*, relative to the genetic variance in the male trait, *G_t_* (Lande 1981). Instability and runaway therefore occur when 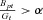, where α is the slope of the line of equilibrium under the model of mate choice we use here (Lande 1981).

We might expect that IGEs contribute more to the conditions favoring runaway than just the direct genetic variances and covariances. Lande (1981) derived the slope 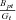 by evaluating 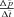. We can similarly derive runaway conditions using equations (5) and (6):


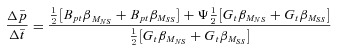
(9)

Simplifying yields conditions for runaway when


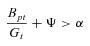
(10)

Expression (10) demonstrates why social environments continue to influence runaway even after covariance is established. Populations perturbed from equilibrium will evolve along lines of constant slope given by 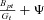. Positive values of Ψ increase the likelihood of runaway sexual selection by conferring steeper slopes (Fig. 2).

Given this key result, it is necessary to evaluate whether IGEs mediated by the social environment alter equilibrium conditions. Substituting selection gradients (first-order derivatives of 3a and 3b) into expressions for the evolution of 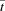 and 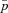 (eqs. 5 and 6) gives:


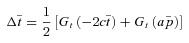
(11)


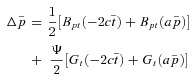
(12)

Setting 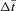 and 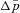 equal to zero defines two isoclines, both of which yield the same line of equilibrium as found in quantitative genetic models of runaway (Mead and Arnold 2004)


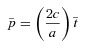
(13)

Thus, while the conditions for runaway are strongly influenced by social environments (eq. 10), equilibrium conditions do not change.

### INTERPRETATION: CONDITIONS FAVORING RUNAWAY

The relationship in equation (10) again highlights the importance of Ψ in sexual selection. A sufficiently large and positive Ψ can cause populations positioned off the line of equilibrium to evolve along a trajectory of slope greater than α even if the covariance between male traits and female preferences is initially absent. As a consequence, the evolution of Ψ itself can make an important contribution to the evolutionary elaboration of a trait. To date, there are only a handful of studies that examine Ψ in any social interactions (Bleakley et al. 2010) and only three that estimate Ψ in the context of sexual selection (Bleakley and Brodie III 2009; Chenoweth et al. 2010; Bailey and Zuk, in press). All three suggest that Ψ can be nonnegative, of substantial magnitude, and subject to evolutionary change. If Ψ is of sufficient magnitude to increase the slope of the population's trajectory above that of the line of equilibrium, the runaway process will begin or continue (Fig. 2). Because female preferences will evolve faster when Ψ is large, the covariance between the male trait and female preference will also develop more readily as this term approaches 1.

Although runaway sexual selection is enhanced by a positive Ψ, a negative Ψ will retard evolution. This result has an appealing intuitive explanation. If social interactions with males dampen the expression of female preferences, Ψ is negative and a larger genetic covariance would be required for runaway. When social environments have a negative effect, we are therefore unlikely to find extravagant sexual ornaments evolving by the Fisher process, which leads to the prediction that we are unlikely to find negative values of Ψ in populations where sexual selection on male traits is strong. In such populations, we might predict that elaboration occurs through male–male competition exclusively. A further prediction also follows: if Ψ varies between populations or habitats, or fluctuates temporally, buildup of genetic covariance between female preferences and the preferred male trait will occur in a patchy manner.

## Including Costs of Preferences

Our initial model provides the simplest starting point for a quantitative genetic model of sexual selection incorporating IGEs generated by the social environment, but it assumes no direct selection on *p*. This corresponds to the initial conditions for sexual selection, where weak preferences reflect a preexisting condition without costs (Fisher 1915, 1958). Once preferences are established and begin to evolve, costs are biologically more realistic if not inevitable. Incorporating costs to mate choice typically results in a point of equilibrium rather than a line of equilibrium for the joint evolution of the trait and preference (Mead and Arnold 2004). However, Fisherian runaway still occurs under a wide range of scenarios where preferences have a cost (Kirkpatrick and Barton 1997; Hall et al. 2000; Houle and Kondrashov 2002). For example, subsequent models have included viability selection on females and different fitness equations (Pomiankowski et al. 1991; Iwasa and Pomiankowski 1995; Pomiankowski and Iwasa 1998). We can extend an influential Iwasa and Pomiankowski (1995) quantitative genetic model of trait–preference coevolution using the IGE framework.

Male traits and female preferences are defined as in equations (1–2b). We modify natural selection on the male trait in line with the Iwasa and Pomiankowski (1995) model, such that natural selection initially incurs very small costs but then rapidly increases with trait elaboration. The male fitness equations become


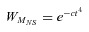
(14a)


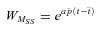
(14b)

Male sexual selection is unchanged and reflects a model of female choice in which the fitness of the male trait depends on the average female preference, plus his deviation from the average male trait. The relative influence of female preference on male fitness is scaled by the constant *a*. The natural selection cost of the male ornament is indicated by *c*. We now also include selection on female preferences


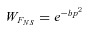
(14c)

where *b* is a parameter of small magnitude describing a cost of female preference.

Finally, mutation bias on the male trait is indicated by *u*, which is the same order of magnitude as *b*.

Joint evolution of the two traits becomes


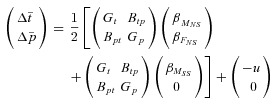
(15)

Genetic variances and the covariance are indicated as *G_t_*, *G_p_*, and *B_tp_*, as before. β_*i*_ indicates selection gradients generated by taking the first-order derivatives of the appropriate fitness equations, evaluated at the population means.

Equilibrium and runaway conditions can now be derived using fast and slow dynamics adapted from population biology applications (Pomiankowski et al. 1991; Iwasa and Pomiankowski 1995). Fast dynamics describe the behavior of evolving populations influenced by parameters of relatively large magnitude, whereas slow dynamics model the behavior when influenced by parameters of small magnitude, which include female preference costs and mutation bias. The fast dynamics can be modeled by disregarding the smaller parameters *b* and *u*, incorporating IGEs, and substituting in the selection gradients. Then, 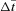 and 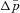 can be written as separate equations:


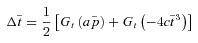
(16)


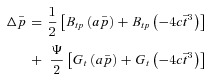
(17)

We evaluate equilibrium conditions under fast dynamics by letting 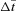 and 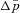 equal zero to define a curve of equilibrium. Rearranging and simplifying gives the curve


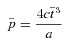
(18)

This is identical to the curve of equilibrium found by Iwasa and Pomiankowski (1995). Runaway sexual selection will occur when the slope of the line of equilibrium, 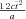, is less than the evolutionary trajectory given by 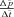. Evaluating the latter using equations (16) and (17) yields runaway conditions when


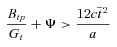
(19)

This inequality is consistent with the result of our main model: Ψ influences the likelihood of instability and runaway, the direction depending on its sign.

Decomposing the behavior of the system into slow dynamics allows us to describe the effects of preference cost *b* and mutation bias *u* on evolutionary dynamics once a population has evolved close to the line of equilibrium. Including a cost to preference collapses the line of equilibrium to a point, and to determine where that point lies it is necessary to derive the per generation change in mate preferences via slow dynamics. This is done by modeling the dynamics of a point close to the line of equilibrium defined by 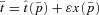, where ɛ represents a constant of the same magnitude of preference cost and mutation bias (Pomiankowski and Iwasa 1993). Substituting into equation (15) to solve for 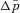



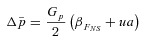
(20)

However, in our case, the expectation or breeding value for *G_p_* is not only the direct additive genetic effects (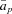) but also includes IGEs contributed by the interacting male phenotype, 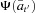. As before, *t*′ is the value of the male trait *t* expressed by the interacting partner. Substituting the first-order derivative of the female fitness function 14c for the selection gradient, plus our expectation for *G_p_* yields


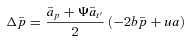
(21)

Change in the male trait at that point only depends on the female preference (Iwasa and Pomiankowski 1995)


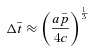
(22)

Evaluating equations (21) and (22) at equilibrium gives the point 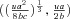, which is identical to the equilibrium found in the model lacking IGEs, and depends only on the constants *a*, *b*, *c* and mutation bias *u*.

Iwasa and Pomiankowski (1995) derive points at which fast dynamics transition to slow dynamics. One key point describes the maximal range of values of 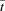 and 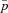 an evolving population can reach before slow dynamics dominate and the population evolves back to the line of equilibrium. This point is given by


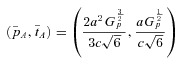
(23)

This transition point in our model becomes


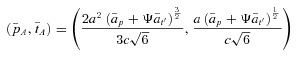
(24)

Positive values of Ψ increase the potential range of trait values that can be reached via the fast dynamics of runaway. Populations can therefore cycle between larger values of 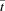 and 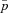 when Ψ is large and positive, and in contrast, they will cycle between more restricted values of 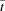 and 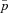 when Ψ < 0.

### INTERPRETATION: PREFERENCE COSTS AND THE EXTENDED MODEL

The extended model describes cyclical coevolution of traits and preferences, where populations displaced from an unstable curve or point of equilibrium evolve under runaway conditions until slow evolutionary dynamics, influenced by smaller parameters describing the costs of female preference and mutation bias on male traits, drive the population back toward equilibrium (Iwasa and Pomiankowski 1995). Social environments clearly affect this dynamic by altering the potential for runaway and either enhancing or diminishing the possible range of traits and preferences the population can reach during cyclical evolution, depending on the precise strength and direction of Ψ.

Taken together, these results indicate that the influence of social environments is consistent when incorporated into sexual selection models that allow costs of choice and mutation bias. In line with these findings, we expect that social environments are unlikely to influence equilibrium conditions in general, but that they will strongly influence the conditions for runaway because the evolutionary trajectory of populations displaced from equilibrium is augmented when Ψ > 0 and retarded when Ψ < 0. IGEs do not affect equilibrium dynamics because selection, not genetics, determines these dynamics (Lande 1981). However, IGEs can substantially impact runaway behavior around those equilibria regardless of the model used, and the stronger the influence and importance of the social environment, the more likely this is to be an unstable rather than a stable equilibrium.

## Discussion

Well before the models of runaway sexual selection in the 1980s (Mead and Arnold 2004), the importance of social interactions above and beyond those involved in the actual act of copulation was acknowledged (see e.g., Bateson 1978; Janetos 1980). Our model expands the social context of sexual selection theory to explicitly acknowledge IGEs that arise from the social environment. Incorporating IGEs into quantitative genetic models of sexual selection shows that in many cases, the potential for runaway sexual selection might be underestimated. Considering socially flexible female choice refines our expectations for the genetic architecture of male ornaments and female preferences, and clarifies the conditions under which we should expect Fisherian runaway to occur. Systems in which male mate choice predominates would be expected to follow similar dynamics.

Four predictions amenable to empirical testing arise from our model. First, IGEs arising from social environments in some cases diminish the requirement for a genetic covariance between female preference and male ornament traits. The need for a relatively strong indirect selection arising from this covariance has been a major theoretical difficulty, because it is not often expected (Kirkpatrick and Barton 1997). Indeed, it is not often observed empirically. The lack of published data on trait–preference covariances might reflect the difficulty of obtaining such measurements, but the fact that covariances have been difficult to detect likewise suggests that they might simply be low in general. Our model provides a way to reconcile the apparent paradox that elaborate sexual ornaments are widely observed while the genetic conditions thought to favor their rapid evolution via the Fisher process are not (Svensson and Gosden 2007). Empirical estimates of the genetic architecture of male ornaments and female preferences, combined with information about the strength of Ψ in the context of interactions around those traits, would help to validate the prediction that trait/preference covariances might be small or negligible in far more cases than previously expected.

Second, social flexibility is expected to be common in populations experiencing strong sexual selection. Large positive values of Ψ increase the likelihood of runaway. Thus, strong social influences on the expression of female mating decisions are predicted when male ornaments are under strong sexual selection. This yields a useful prediction about when we should expect to observe strong social flexibility in mate choice in natural systems, which can be tested by relating variation in Ψ to the strength of sexual selection on male ornaments, using either intraspecific or interspecific comparisons.

Third, heterogeneity in Ψ across populations or species is expected to contribute to more rapid rates of divergence. Even when the distribution of female preference values is homogeneous and static, evolution via sexual selection will occur at different rates and can lead to divergence on a relatively fine scale if social environments vary spatially (Agrawal et al. 2001; Gosden and Svensson 2008). Such variation can play an important role in setting up divergent sexual selection pressures in populations that are subdivided or otherwise experience restricted gene flow. This prediction can be tested by relating variation in Ψ to the strength and direction of sexual trait divergence and reproductive isolation among populations. We know that Ψ can evolve (Chenoweth et al. 2010). Empirically, genetic heterogeneity among populations in terms of interacting partners influences mating in *Drosophila* (Krupp et al. 2008), and heterogeneity in social flexibility in female choice among geographically isolated populations of the cricket *T. oceanicus* results in differing values of Ψ, which range from approximately –0.6 to 0.4 (Bailey and Zuk, in press). Laboratory studies would further resolve the causal relationship; for example, by using inbred strains (Bleakley and Brodie III 2009), artificial selection (Chenoweth et al. 2010), or mixed isofemale or mutant lines (Kent et al. 2008; Krupp et al. 2008) to manipulate the social environment and thereby Ψ, and assess knock-on effects on the Fisher process. For example, mate choice learning has been implicated in population divergence and reinforcement (Servedio et al. 2009; Svensson et al. 2010), and IGEs arising through social interactions inherent in learning about the social environment could generate the evolutionary dynamics observed in our model if they affect the evolution of traits under sexual selection. Variation in learned mate preferences, and therefore Ψ, among isolated populations or experimental lines is likely to exist in many tractable systems, including *D. melanogaster* (Dukas 2005), damselflies (Gosden and Svensson 2008), and guppies (Magurran and Ramnarine 2004).

Finally, our model predicts that traits displayed in a social setting should experience greater elaboration via the Fisher process because of the IGEs arising from flexible female preferences. The comparative method provides a framework for testing this, for example by examining an assemblage of insect species showing variation in the degree of social interaction prior to mating, and relating it to variation in male ornament elaboration. Systems in which the secondary sexual traits of multiple taxa are well characterized, for example *Drosophila* spp. or some passerine groups, might provide fertile ground for empirical testing.

In conclusion, our model highlights the importance of social flexibility in female preferences, a topic that has attracted considerable attention in the literature but surprisingly few theoretical treatments (Widemo and Sæther 2005). Flexibility in mate choice can be caused by numerous mechanisms that have attracted the attention of researchers, so the model presented here has a potentially wide application. However, not all social influences will have the same effect: when flexibility arising from social environments opposes selection on female preferences through genetic covariance, the genetic architecture of male traits and female preferences may fall short of what is needed to initiate or sustain runaway. To test the prediction that the opportunity for trait/preference elaboration covaries with Ψ, it is necessary to know in what systems and under what circumstances flexibility in female choice translates to a negative or a positive Ψ. Empirical measures of both IGEs and Ψ in the context of sexual selection are needed.
